# Seeking biomarkers for acute graft-versus-host disease: where we are and where we are heading?

**DOI:** 10.1186/s40364-019-0167-x

**Published:** 2019-08-07

**Authors:** Xiao-Su Zhao, Xiao-Jun Huang

**Affiliations:** 1 Peking University Peopl’s Hospital, Peking University Institute of Hematology, No.11 Xizhimen South Street, Beijing, 100044 China; 2 National Clinical Research Center for Hematologic Disease, Beijing, China; 3 Beijing Key Laboratory of Hematopoietic Stem Cell Transplantation, Beijing, China; 4 Peking-Tsinghua Center for Life Sciences, Beijing, China

**Keywords:** Acute graft-versus-host disease, Biomarker

## Abstract

Acute graft-versus-host disease (aGVHD) is one of the most important complications after allogeneic hematopoietic stem cell transplantation (allo-HSCT), which would seriously affect the clinical outcomes of patients. Early diagnosis and early intervention are keys for improving its curative efficacy. Thus, seeking the biomarkers of aGVHD that can accurately identify and diagnose aGVHD is very important to guiding the intervention and treatment of aGVHD. For the past decades, many studies have focused on searching for aGVHD-related biological markers to assist in diagnosis, early warning, and risk stratification. Unfortunately, until now, no reliable aGVHD biomarker is available that is recognized and widely used in clinical practice. With the continuous development of biological technology, as well as our in-depth understanding of the pathophysiologic mechanism of aGVHD, the selection, examination and application of biological markers have changed much. In this review, we summarized the progress of aGVHD biological marker screening, identification, preliminary clinical application, and look forward to a promising development direction in the future.

## Introduction

Acute graft-versus-host disease (aGVHD) is one of the most important complications after allogeneic hematopoietic stem cell transplantation (allo-HSCT) that seriously affects the prognosis of patients. Early diagnosis and early intervention are keys to improving its curative efficacy. Thus, biological markers must be identified that can accurately identify and diagnose aGVHD. For nearly 20 years, many studies have focused on searching for aGVHD-related biological markers to assist in diagnosis, early warning, and risk stratification; however, until now, no reliable aGVHD biomarker is available that is recognized and widely used in clinical practice. With the continuous development of biological technology, as well as our in-depth understanding of the pathophysiologic mechanism of aGVHD, people searching for aGVHD biomarkers must continue to explore, expand and progress. We review the progress of aGVHD biological marker screening, identification and preliminary clinical application and look forward to a promising development direction in the future.

## Ideal biomarker for aGVHD

An ideal biomarker should to identify a certain disease sufficiently early and accurately and should potentially guide the appropriate preemptive treatments. Based on their different roles during the course of disease, the National Institutes of Health-sponsored working group has divided biomarkers into four types for diagnosis, prognosis, prediction, and response evaluation. Some biomarkers can serve as panmarkers covering the whole course of disease, such as REG3α and stimulation 2 (ST2) [1–3], but some can only be used in certain aspects. For example, hepatocyte growth factor (HGF) is used in the diagnosis of liver aGVHD [4], while CD146+ T cells and invariant natural killer T cells can only be employed in the prognosis of aGVHD [5, 6]. According to the clinical characteristics of aGVHD, an optimal biomarker should meet the following criteria: [1] satisfactory sensitivity and specificity for detecting aGVHD; specificity is particularly important when distinguishing aGVHD from other posttransplant comorbidities; [2] samples for testing can be obtained in a relatively noninvasive way; [3] fast, economical and reproducible methods can be used for detection; and [4] the biomarker level should be related to the severity, curative effect, and prognosis of aGVHD.

## Exploratory studies of aGVHD biomarkers

Considering the research history of aGVHD biomarkers, these potential markers are divided into two main categories according to the time of transplantation: predetermined and dynamic markers. Determined biomarkers refer to factors that have been defined before transplantation, such as by donor graft composition or inflammatory- or immune-related gene polymorphism. However, dynamic markers refer to factors that could be monitored after transplantation and the expression at certain time points might predict the occurrence or severity of aGVHD. These biomarkers include many cytokines, inflammatory chemotactic factors, vascular endothelial-related factors and target organ injury-related proteins.

### Predetermined biomarkers of aGVHD

#### Donor graft composition

Because the mechanism of aGVHD is mainly via the attack of donor T cells on host target organ tissue [7], many studies focus on the effect of donor T cells/different functional subsets on aGVHD during transplantation. For example, previous studies have shown that a lower frequency of infused naïve CD8+ T cells is associated with a reduced risk of aGVHD [8, 9]. It was also demonstrated that the ratio of CD4/CD8 in transfused bone marrow (BM) is related to the occurrence of aGVHD. Luo et al. reported that BM grafts with CD4/CD8 ratios ≥1.16 are associated with a significantly increased risk of grades II to IV aGVHD [10]. A recent in vitro mixed lymphocyte culture experiment also supported this conclusion, in which the CD4/CD8 ratio shifted toward an increase in CD4+ T cells and a decrease in CD8+ T cells in the GVHD group [11]. Darius Sairafi et al. reported that, compared with the patients without aGVHD, those who underwent aGVHD showed higher frequencies of CD8+ and CD69+ T cells in the graft [11]. Another T-cell subtype are γδ T cells, which, in contrast to allogeneic αβ T cells, are not alloreactive and do not induce aGVHD [12]. Pabst et al. investigated the effect of γδ T cells in peripheral blood stem cell (PBSC) grafts on clinical outcomes after allo-HSCT. They found that, in patients receiving a higher frequency of donor γδ T cells in the grafts, the incidence of grade II-IV aGVHD was much higher [13]. However, another study reported an obvious decrease in the percentage and number of γδ T cells in patients with aGVHD [14]. Thus, the effects of graft components on the occurrence of aGVHD are gradually refined into various subtypes of T cells. Their impact on aGVHD was correlated with the conditioning regimen, transplant type, amount of infused cells, and proportion of other components in the graft.

Regulatory T cells (Tregs), as the main cell subsets that play an immunosuppressive role, are also a focus. It was reported that the low numbers or proportions of Tregs in peripheral blood are associated with aGVHD [15, 16]. CD69 has always been regarded as one of the earliest markers emerging after T-cell activation [17]. However, in the past few years, studies have also shown that special types of CD69+ T cells might serve as a group of regulatory T cells [18, 19]. Lu et al. further confirmed this phenomenon in a human study, demonstrating that a high frequency and increased numbers of CD4 + CD25-CD69+ T cells are associated with a reduced risk of aGVHD [20]. More recently, a few other T-cell subsets have also been proven to contribute to the progression of aGVHD. For instance, although comprising only a small number of T cells, invariant natural killer T cells (iNKT cells) have potent immunomodulatory functions. Several studies have demonstrated that iNKTs can attenuate aGVHD [21, 22]. Chaidos et al. reported that the use of peripheral blood stem cell (PBSC) grafts with fewer CD4- iNKTs is associated with an increased risk of aGVHD [5]. Florent Malard et al. also reported that the number of iNKTs contained in PBSC grafts was the only factor with a significant impact on the outcome after allo-HSCT [23]. Moreover, iNKTs have also been shown to suppress aGVHD and display anti-leukemia effects. Although iNKTs may be a promising biomarker of aGVHD, challenges are expected in their clinical use due to the very limited number in peripheral blood. They are difficult to detect by flow cytometry more accurately, especially at different centers.

Accumulating studies in recent years have also found that, in addition to the above T-cell subsets, other subgroups of cells are more or less involved in the pathologic process of aGVHD, with functions such as immune regulation, such as myeloid derived suppressor cells (MDSCs) and regulatory B cells (Bregs). MDSCs have been reported as major immunosuppressive cells in chronic inflammation, infection, cancer, autoimmune disease and aGVHD [24, 25]. Granulocyte-colony stimulating factor (G-CSF)-mobilized MDSCs are closely associated with GVHD in allo-HSCT [26]. Antonio et al. reported that G-CSF-induced expansion of M-MDSCs (Lin^low/neg^HLA-DR − CD11b + CD33 + CD14+) is the only graft factor to predict aGVHD [27]. Similarly, we found that patients who received a higher absolute count of monocytic MDSCs (M-MDSCs) or promyelocytic MDSCs (P-MDSCs) exhibited a lower incidence of grade II-IV aGVHD in 62 patients who underwent haplo-identical HSCT [28]. Bregs are a newly described subset of B cells that plays important roles in autoimmunity [29–32]. In a mouse model, Hu et al. found that the cotransplantation of donor-derived Bregs could result in a significant shift from Th1 + Th17 to Th2. Additionally, the cotransplantation of donor-derived Bregs can increase the percentages of Tregs in the target organs of aGVHD, such as the skin, intestine, colon, liver, and lung [33]. A German study demonstrated that host and donor B cell-derived IL-10 provides a unique mechanism of suppression of acute GVHD in a mouse model [32]. However, whether Bregs in grafts could predict the occurrence of human aGVHD needs confirmation in clinical studies. Additionally, these cell subsets also lack recognized phenotypic markers; thus, they cannot be universally employed in the clinic.

#### Gene polymorphism of cytokines

aGVHD is a complex pathologic process that involves many cytokines, including interleukin-1(IL-1), IL-6, tumor necrosis factor α (TNFα), interferon-γ (IFN-γ) and IL-2. In addition to the HLA genes, the genetic differences between transplant recipients and donors also play important roles in aGVHD [34]. In the past ten years, increasingly more single-nucleotide polymorphisms (SNPs) of genes related to innate and adaptive immune responses have been identified [35–37]. It was found that several genes regulate the function of immune cells, their receptors, cytokines, and chemokines and effector molecules, which are involved in the classic ‘cytokine storm’ of aGVHD [35–41]. Although many studies reported that different SNPs are associated with the risk of aGVHD, most of their results have been difficult to replicate in different cohorts, making their use impractical. With the development of sequencing technology, a study used the genotyped and SNP data from genome-wide scans of 1,298 allo-HSCT donors and recipients to investigate the genetic associations with aGVHD. They found obvious and confirmed associations with the IL-6 SNP genotype in both the patient and donor genomes, as well as with the aGVHD phenotypes. This significant association might be interpreted by the SNP rs 1800795 of IL-6 being located at the position that might result in the upregulation of IL-6 gene transcription and higher circulating IL-6 expression levels [42]. However, this association only refers to the SNP of a single cytokine.

Thus, a few studies began to focus on establishing a model comprising several cytokine genes with SNPs by statistical methods to predict the occurrence of aGVHD. Alam N et al. have built a risk model incorporating donor IL-6 and the IFN-γ genotype and demonstrated that it could identify patients at high risk of SR aGVHD with the donor genotypes of IL6 (rs1800797) and IFN-γ (rs2069727) along with the gastrointestinal involvement of aGVHD [43]. More recently, a study including 25 SNPs in 12 cytokine genes was performed to evaluate the risk of aGVHD. Their results showed that models including clinical and genetic variables predicted severe aGVHD much better than models including only clinical variables or only SNPs of cytokines [3]. Thus, the genetic data of cytokines combined with other clinical parameters might provide more efficient predictive tools for aGVHD. However, these cytokines are often closely related to inflammatory processes and are not very specific factors for GVHD. Although we can examine their polymorphisms, considering the complexity of posttransplant complications, the efficiency to predict aGVHD by the SNPs of these cytokines and the possibility of excluding other diagnoses have yet to be explored.

### Dynamic monitoring of the expression of aGVHD biomarkers

#### Single parameters as biomarkers

Considering the research history of aGVHD biomarkers, initial studies focused on identifying individual inflammatory cytokines, chemokines and other molecules that can early warn and assist the diagnosis of aGVHD. Dynamic monitoring of changes in the expression levels of these key factors after transplantation, especially before the occurrence of aGVHD (from + 3 days to + 14 days after transplantation), is considered to play an important role in the early warning of aGVHD.

##### Cytokines related to T cells

Many studies have been performed on several key inflammatory factors, including IL-2, TNFα, TGFβ, IL-7 and IL-15, during the pathological process of aGVHD. For example, studies in Japan and Germany have concluded that the detection of soluble IL-2 receptor (sIL-2R) levels at + 3 days after transplantation can predict the occurrence of aGVHD [44], and its level is closely related to the severity of aGVHD. Other studies have also indicated that a significant increase in sIL-2R could be observed 1–2 weeks before the onset of aGVHD [45, 46]. Similar to IL-2, the level of serum TNFα and its receptor, TNFR1, is also associated with aGVHD [47, 48]. However, these two key factors in aGVHD face the same challenge that they are not specific to aGVHD. Under various inflammatory conditions, they will also be present during infection, veno-occlusive disease and pulmonary toxicity. Similarly, several Th1 inflammatory factors, such as IFNγ, IL-18, and Th2 factors IL-10 and TGFβ, have also been proven to be associated with aGVHD. Two studies showed that patients with aGVHD had high levels of IL-18 that strongly correlated with the severity of aGVHD [49, 50]. However, the cause might be due to the negative feedback that some Th2 cytokines are also elevated in aGVHD. For example, early after allo-HSCT, donor T cells were the predominant source of TGFβ and could prevent aGVHD; additionally, a Polish study found that, in patients with aGVHD, the mRNA expression of TGFβ and its serum concentration remained low until day + 30 after transplantation compared with those at the day of transplantation [51]. A similar result was observed for another Th2 cytokine, IL-10. Weston LE et al. have shown that a high frequency of donor cells producing IL-10 was correlated with the absence of aGVHD after allo-HSCT, while a low frequency of these cells was strongly associated with severe aGVHD [52].

IL-7 can expand donor T cells and promote immune reconstitution. Poiret T et al. showed that low soluble IL-7 receptor (sIL-7R) was associated with any grade of aGVHD at 2 and 6 months after transplantation and it was an independent risk factor associated with aGVHD [53]. Thiant S et al. investigated the relationship between the plasma level of IL-7 & IL-15 and aGVHD. They found that only the IL-7 level measured at + 30 d was the foremost predictive factor for grade II-IV aGVHD, while IL-15 was not but acted more such as a general inflammatory marker [54].

ST2, which is the receptor of IL-33, was first discovered using therapy-resistant aGVHD samples by high-resolution mass spectrometry [2]. Until now, ST2 is considered one of the most validated biomarkers of aGVHD, as well as non-relapse mortality (NRM), whether measured alone or with other biomarkers [55]. Mark T. Vander Lugt et al. compared 12 biomarkers in plasma approximately 16 days after the initial treatment of aGVHD from patients with complete remission or progressive aGVHD. They found that ST2 had the most significant association with resistance to aGVHD therapy. Based on this result, they indicated that ST2 levels measured at the initiation of treatment of aGVHD within the first month after allo-HSCT would improve the risk stratification for refractory aGVHD. Although high ST2 levels predicted the higher mortality of aGVHD, nonspecific tissue damage or the genetic background can also affect its plasma expression [56].

##### Molecules related to tissue/cell injuries

In addition to the above inflammatory cytokines and their receptors, tissue/cell injury-related molecules have also been studied as potential biomarkers of aGVHD. HGF is a circulating molecule that is related to tissue repair after injury. Okamoto T et al. reported that the serum concentrations of HGF in transplanted patients without aGVHD were consistently low, while those in patients with acute GVHD increased with exacerbation [57]. Another molecule, follistatin, which is expressed in many tissues, including endothelial cells, skeletal muscle and brain, functions in tissue inflammation and repair. It works as an angiogenic factor in migrating endothelial cells to heal wounds [58, 59]. A previous study confirmed its association with the occurrence of aGVHD and subsequent survival. Additionally, plasma follistatin was elevated at the onset of aGVHD, and follistatin levels > 2000 pg/ml in patients at day 28 after treatment with aGVHD are an independent risk factor for overall survival [60]. Furthermore, a large cohort study from Minnesota demonstrated that the follistatin levels of both transplant recipients and donors were associated with the incidence of aGVHD. The increased follistatin levels on day 28 were correlated with the onset of grade II-IV aGVHD prior to + 28 d [61]. Similarly, angiopoetin-2 (ANG2) is an endothelial factor involved in the pathogenesis of aGVHD [62]. The kinetics of T-cell activation markers and molecules of endothelial dysfunction in serum of patients with sensitive and refractory aGVHD was determined. Finally, they found that endothelial cell vulnerability and dysfunction, rather than refractory T-cell activity, guides the therapy of refractory aGVHD [63].

In recent years, the study of aGVHD biomarkers has gradually focused on the molecules associated with end target organs of GVHD, which may be more specific than previous inflammatory factors. Thus, aGVHD and other posttransplant complications can be distinguished.

Elafin was initially identified as an elastase inhibitor highly expressed in the inflamed epidermis, which is an alarm antiprotease secreted in response to IL-1 and TNFα [55, 64, 65]. Because it is produced by GVHD target keratinocytes rather than by the effector cells capable of injury to all GVHD target organs, elafin has been considered an aGVHD-specific biomarker [7]. Sophie Paczesny et al., in 2010, had first proven that the plasma level of elafin is significantly higher at the onset of skin GVHD and is correlated with the eventual maximum grade of aGVHD [66]. Subsequently, an Austrian study confirmed that aGVHD and drug hypersensitivity rashes displayed different degrees of elafin levels. They also demonstrated that elafin-high aGVHD lesions presented with epidermal thickening and were associated with poor clinical outcomes [12]. More recently, Mahabal GD et al. indicated that tissue elafin is a useful immunohistochemical marker for skin aGVHD, and the sensitivity and specificity of elafin immunohistochemistry to predict acute skin GVHD were 100 and 75%, respectively [67]. Although the biomarker of skin aGVHD, elafin, is more specific, most skin aGVHD is easy to control, and the incidence of associated mortality is very low. Thus, the necessity for clinical application of this indicator is challenged.

In acute intestinal GVHD, apoptotic intestinal crypt cells were often regarded as a characteristic feature. The serum level of cytokeratin-18 fragments (CK18F), the degradation product, was correlated with acute hepato-intestinal GVHD. More recently, Sandra Sauer et al. found that both total CK18 and apoptotic CK18F increased at the onset of aGVHD. For those with clinical acute hepato-intestinal GVHD, total CK18 would already be significantly increased 7–14 days before clinical symptoms [68]. They indicated that total CK18 had the highest sensitivity and specificity for patients with proven intestinal and hepatic GVHD. Additionally, their previous study also showed that CK18F serum levels could increase prior to the clinical symptoms of aGVHD [69]. Finally, their team suggested that weekly monitoring of the total CK18/CK18F levels within the first month after allo-HSCT might be a promising and efficient way to warn of acute hepato-intestinal GVHD.

Regarding gastrointestinal tract aGVHD, regenerating islet-derived 3-α (REG3α), which is considered a very efficient biomarker, is an antimicrobial protein expressed in Paneth cells. First, Ferrara JL et al. reported that the level of plasma REG3α was 3-fold higher in patients at gastrointestinal GVHD onset than in all other patients and could also predict the response to therapy at 4 weeks and 1-year of NRM [1]. Andrew C. Harris et al. showed that, compared with the other two biomarkers of aGVHD, HGF and CKF18, REG3α is the more promising marker of lower gastrointestinal aGVHD [4].

Although monitoring of these biomarkers after transplantation has certain predictive significance for aGVHD, the meaningful detection time points of different markers are not the same. Therefore, their clinical application is restricted.

#### Multiparameter panel of biomarkers

Although various molecules/proteins have been found to be associated with aGVHD, until now, no reliable single marker could be employed clinically. aGVHD is a rapidly occurring complication similar to an inflammatory response in the early stages of transplantation, causing cytokine storms involving various cytokines. Additionally, many chemotactic factors and target organ-related molecules participate in the pathological process of aGVHD. Therefore, in recent years, researchers have gradually noticed that the efficacy of single factors for the early warning and diagnosis of the occurrence of aGVHD is limited, and it is difficult to distinguish it from other inflammatory complications. Thus, they began to try to reference a group of molecules using unbiased as well as targeted proteomic technology to predict and diagnose the occurrence and prognosis of aGVHD.

##### Plasma biomarker panels of aGVHD

One famous study about the biomarker panel of aGVHD came from the University of Michigan. They screened plasma samples from patients who received allo-HSCT with an antibody microarray for approximately 120 proteins and found 8 potential proteins for the diagnosis of aGVHD. They then validated these potential markers using enzyme-linked immunosorbent assay (ELISA) in a large cohort of patients. Finally, they determined a biomarker panel composed of 4 proteins (IL-2Rα, TNFR1, IL-8 and HGF) that could confirm the diagnosis of GVHD in patients at the onset of clinical symptoms of GVHD and provide prognostic information independent of GVHD severity [70]. Subsequently, the same group also used a composite biomarker panel (IL-2Rα; TNFR1; HGF; IL-8; elafin, a skin-specific marker; and REG3α, a gastrointestinal tract–specific marker) to discriminate between therapy responsive and nonresponsive patients who received aGVHD treatment. They measured the levels of samples obtained at the onset of treatment, + 14 d, and + 28 d in a multicenter, randomized, 4-arm phase 2 clinical trial about aGVHD. The results showed that, at each of these three time points, this 6-protein biomarker panel can be used for the early identification of patients at different risks for refractory aGVHD or death [71]. Likewise, the data from a multicenter clinical trial showed that an established a biomarker algorithm (ST2, REG3α, TNFR1, and IL-2Rα) based on a blood sample of + 7 d after allo-HSCT could consistently identify the patients at high risk for lethal aGVHD and nonrelapse mortality [72].

##### Urinary and salivary biomarkers of aGVHD

Although peripheral blood specimen testing is relatively noninvasive compared with tissue biopsy, it still requires the drawing of blood from patients for examination. More importantly, the components of plasma are more complicated than those of urine and are relatively unstable. Thus, some studies have begun to investigate whether other, more noninvasive tests can assist in the early warning and diagnosis of aGVHD. Weissinger et al. have identified potential peptide biomarkers in the urine samples of HSCT patients using capillary electrophoresis and tandem mass spectrometry. The 17-peptide panel developed from the data of this study (aGVHD_MS17) could accurately recognize aGVHD-positive patients before the general clinical diagnosis. aGVHD_MS17 positivity was the only strong predictor for grade III-IV aGVHD in multivariate regression analysis. This aGVHD_MS17 was derived from albumin, β2-microglobulin, CD99, fibronectin and various collagen α-chains, indicating inflammation, activation of T cells and changes in the extracellular matrix as signs of aGVHD-induced organ injury [73]. Another highly attractive specimen is whole saliva for noninvasive examination. Saliva includes organic and inorganic solutes and some peptides and proteins related to both innate and adaptive immunity whose levels would be abnormal in patients with autoimmune disease [74]. Thus, some researchers believe that these changes might also be seen in patients with GVHD. Chiusolo P et al. collected saliva specimens from 40 consecutive patients who received allo-HSCT and used high-performance liquid chromatography combined with electrospray-ionization mass spectrometry for analysis. The results showed that two saliva proteins, S100A8 and S100A9, might be biomarkers of aGVHD [75]. However, this conclusion still needs further studies to clarify the role of these proteins as a marker of GVHD or as an index of mucosal inflammation.

The study of aGVHD biomarkers has been gradually transferred to the era of multifactor combined diagnosis, which is brought about by multiomics. More attention is paid to the role of damage markers (tissues, epithelial cells, and blood vessels) in the end target organs of aGVHD, replacing single inflammatory factors with lower specificity in the diagnosis. In summary, the pre-emptive therapy of aGVHD might be more specific and efficient only if the panel of biomarkers employed includes the biomarkers playing essential roles in the pathophysiology of aGVHD.

### Discovery and identification of a new generation of biomarkers: gradually strengthening the concept of a “spectrum”

#### MicroRNAs

MicroRNAs represent noncoding RNAs that direct a complex network of translational repression. MicroRNAs can simultaneously regulate multiple genes or can be regulated by multiple genes and participate in multiple biological processes. Importantly, their expression could change before the changes in protein levels. During the past several years, many studies have reported the strong associations between certain microRNAs and important cytokine pathways related to aGVHD. These biological traits make microRNAs potential biomarkers of disease-specific spectrum changes. One of the most famous microRNAs associated with aGVHD is microRNA-155. MicroRNA-155 has been shown to be upregulated during T-cell activation [76]. A previous study demonstrated that the knockout of microRNA-155 in dendritic cells could protect against aGVHD by limiting the activation of molecular networks or inflammation that responds to the pathogens or DAMPs [77]. Its upregulation was shown in specimens from patients with pathologic evidence of intestinal aGVHD and might be a novel target for GVHD treatment. Another example is microRNA-146a, which interacts with TNF receptor-associated factor 6 (TRAF6) and regulates TNFα [78]. Similarly, Zhao et al. showed that the elevated plasma miR-153-3p levels at + 7 d after transplantation could be used to predict aGVHD. Additionally, they proved that microRNA-153-3p participates in aGVHD development by inhibiting indoleamine-2,3-dioxygenase (IDO) expression [79]. Furthermore, Xiao B et al. developed a model including 4 microRNAs (microRNA-423, microRNA-199a-3p, microRNA-93, and microRNA-377) that can predict the probability of aGVHD. They have identified a specific plasma microRNA signature that may serve as an independent biomarker for the prediction, diagnosis, and prognosis of aGVHD [80]. Thus, given that microRNAs have tissue specificity and stability in many body fluids, they have received increased attention about their potential roles as minimally invasive biomarkers for aGVHD. However, the testing and standardization of microRNAs remain the major obstacles to preventing them from becoming practical aGVHD biomarkers.

#### Extracellular vesicles

Recently, extracellular vesicles (EVs) have become a promising new type of biomarker in various diseases, including aGVHD. They can be secreted by many types of cells and play an important role in the secretion of soluble factors such as cytokines, growth factors, chemokines and hormones. Like microRNAs, they can be extracted from body fluids noninvasively, makes them very attractive for diagnostic applications. G Lia et al. observed a correlation among three potential biomarkers expressed on EV surface and aGVHD onset using flow cytometry. Their data indicated that CD146 was associated with an increased risk of developing aGVHD (~ 60%), whereas CD31 and CD140-α was associated with a decreased risk—by almost 40 and 60%, respectively. Although this conclusion needs prospective studies to confirm, their preliminary findings highly encourage the investigators about the function of EVs in aGVHD [81].

#### Intestinal microbiome

The gastrointestinal microbiome is involved in vital biological functions, including the maintenance of immune homeostasis and modulation of intestinal development [82]. The gastrointestinal tract is one of the target organs of aGVHD. Accumulating evidence has indicated that the microbiota and their metabolites could help build the immune system and alter the host susceptibility to aGVHD [83, 84]. In recent years, rapid developments of molecular techniques have facilitated the extensive exploration of the gastrointestinal microbial system. Now, next-generation sequencing (NGS) technologies, such as 16S rDNA sequencing and metagenomics, can help us further identify and recognize microorganisms that cannot be cultured. Thus, microbiota would be a new warning biomarker of intestinal aGVHD in the near future and another reflection of a disease-related “spectrum”. The studies of Memorial Sloan Kettering Cancer Center demonstrated that elimination of Lactobacillales before allo-HSCT would aggravate aGVHD in mice, and similar microbiome patterns were also seen in patients during the onset of intestinal aGVHD [83]. The phenomenon that the bacterial diversity is decreased in aGVHD patients has been reported in several studies [83–85]. Moreover, studies from the same group showed reduced aGVHD lethality in patients harboring increased amounts of bacteria belonging to the genus *Blautia*, the higher abundance of which was correlated with improved survival [85]. Previous studies also found posttransplant monodomination of the intestinal microbiome with *Enterococcus* spp*.* at different centers that was significantly related to severe aGVHD [84, 86]. Another study from the University of Texas Southwestern Medical Center showed that obvious depletion of anti-inflammatory *Clostrtidia* spp. (AIC) could be found prior to the occurrence of aGVHD in pediatric patients after allo-HSCT [87]. According to the above advances, fecal microbiota transplantation (FMT) in recent years has also been gradually used in the clinic for aGVHD treatment. More recently, some promising data from clinical trials have been published and confirmed the feasibility and efficacy in microbiome diversity improvement in transplant patients [88, 89]. However, an increasing number of studies has confirmed that intestinal flora is closely related to many disease processes [90–93]. Additionally, the current sequencing method based on 16S rRNA needs to be standardized and assisted by experienced biological information professionals. Thus, there is still a long way to go before routine clinical application.

## Prospects

Although several biomarkers/combinations have been used in the exploration of clinical trials, no reliable aGVHD biomarker is currently available that can be widely applied to clinical practice due to poor repeatability. aGVHD develops quickly, and its pathological process is very complicated, which could be influenced by many factors. The current existing biomarkers of aGVHD developed by previous studies still have the following drawbacks, thus restricting their clinical application. 1. **Specificity**: they are single parameters, such as inflammatory cytokines, thus lacking specificity. 2. **Clinical utility**: different biomarkers may have predictive value toward different target organs of aGVHD and need to be examined at various determined time points after HSCT. Thus, it would be difficult to bring these indicators together for clinical early warning. 3. **Application and standardization:** some new biomarkers need to be measured by using new technology, including next-generation sequencing, iTRAQ & label-free MS approaches, as well as sophisticated big data analytics. Before applying these biomarkers clinically, these methods should be generally stable, and the cost of such methods must allow most patients to afford treatment. Therefore, future studies seeking potential biomarkers of aGVHD may focus on the following areas: 1. Due to the real-world heterogeneity in patients undergoing HSCT, screening should be performed in a more homogeneous population (e.g., the same type of transplantation, conditioning regimen, and disease status). Achieving verification from multicenters is feasible only if strict conditions are set. This approach allows us to obtain some biomarkers with clearly predictive values, which will be easy to apply for certain patient groups. 2. It would be more valuable to explore the biomarkers associated with severe intestinal and hepatic aGVHD for risk stratification and guiding treatment. 3. How to determine the opportune moment of examination according to the clinical features of aGVHD remains to be answered. 4. We should use a standardized method to examine the discovered biomarkers and fulfil the validation. 5. With the current era of big data, the future of aGVHD risk prediction research may focus on machine learning, utilizing a full range of big-data deep analysis and including the construction of new clinical & laboratory multiparameter mathematical models, rather than only studying a few biological markers. 6. Finally, the question that may not have previously been of concern: how can we design appropriate clinical intervention based on these reliable biomarkers or predictive models to improve the prognosis of patients.

## Data Availability

Not applicable.

## Abbreviations

aGVHD: Acute graft-versus-host disease
allo-HSCT: Allogeneic hematopoietic stem cell transplantation
Bregs: Regulatory B cells
CK18F: Cytokeratin-18 fragments
ELISA: Enzyme-linked immunosorbent assay
EVs: Extracellular vesicles
G-CSF: Granulocyte-colony stimulating factor
HGF: Hepatocyte growth factor
IDO: Indoleamine-2,3-dioxygenase
IFN-γ: Interferon-γ
IL-1: Interleukin-1
iNKT cells: Invariant natural killer T cells
MDSCs: Myeloid derived suppressor cells
NGS: Next-generation sequencing
NRM: Non-relapse motality
PBSC: Peripheral blood stem cell
REG3α: Regenerating islet-derived 3-α
sIL-2R: Soluble IL-2 receptor
SNPs: Single-nucleotide polymorphisms
ST2: Stimulation 2
TNFα: Tumor necrosis factor α
TRAF6: TNF receptor-associated factor 6
Tregs: Regulatory T cells
